# The Outcomes and Prognostic Factors of the Very Elderly Requiring Prolonged Mechanical Ventilation in a Single Respiratory Care Center

**DOI:** 10.1097/MD.0000000000002479

**Published:** 2016-01-15

**Authors:** Chih-Cheng Lai, Shian-Chin Ko, Chin-Ming Chen, Shih-Feng Weng, Kuei-Ling Tseng, Kuo-Chen Cheng

**Affiliations:** From the Department of Intensive Care Medicine, Chi Mei Medical Center, Liouying (C-CL); Department of Internal Medicine (S-CK, K-LT, K-CC); Intensive Care Medicine (C-MC); Medical research, Chi Mei Medical Center, Tainan(C-MC); Department of Healthcare Administration and Medical Informatics, Kaohsiung Medical University, Kaohsiung (S-FW); and Department of Safety Health and Environmental Engineering, Chung Hwa University of Medical Technology, Tainan, Taiwan (K-CC).

## Abstract

This study investigated the outcomes and the prognostic factors among the very elderly (patients ≥80 years old) requiring prolonged mechanical ventilation (PMV).

Between 2006 and 2014, all of the very elderly patients of age 80 or more transferred to respiratory care center (RCC) of a tertiary medical center were retrospectively identified, and only patients who used mechanical ventilation (MV) for >3 weeks were included in this study.

A total of 510 very elderly patients undergoing PMV were identified. The mean age of the patients was 84.3 ± 3.3 years, and it ranged from 80 to 96 years. Male comprised most of the patients (n = 269, 52.7%), and most of the patients were transferred to RCC from medical ICU (n = 357, 70.0%). The APACHE II scores on RCC admission was 17.6 ± 6.0. At least 1 comorbidity was found in 419 (82.2%) patients. No significant differences of gender, disease severity, diagnosis, dialysis, laboratory examinations, comorbidities, and outcome were found between octogenarians (aged 80–89) and nonagenarians (aged ≥ 90). The overall in-hospital mortality rate was 21.8%. In the multivariate analysis, patients who had APACHE II score ≥ 15(odds ratio [OR], 2.30, 95% confidence interval [CI], 1.36–3.90), or albumin ≤ 2 g/dL (OR, 3.92, 95% CI, 2.17–7.01) were more likely to have significant in-hospital mortality (*P* < 0.05).

The in-hospital mortality rate of the very elderly PMV patients in our RCC is 21.8%, and poor outcomes in this specific population were found to be associated with a higher APACHE II score and lower albumin level.

## INTRODUCTION

Along with the increasing populations of aging and patients with multiple comorbidities, the increasing availability of mechanical ventilation, and the improvement of critical care, more and more patients may survive the early stage of acute respiratory failure and further require prolonged mechanical ventilation (PMV).^1–3^ PMV, defined as those who use MV for at least 6 h daily for consecutive 21 days or more, is always associated with high cost and intensive-labor. To effectively use this advance medical technology and reduce the heavy utilization of intensive care unit (ICU), several Western countries had established respiratory intensive care units or facilities specifically for respiratory care.^4,5^ In Taiwan, there is no exception. In 2000, Taiwan Bureau of National Health Insurance (BNHI) implemented an integrated prospective payment program (IPP) for caring patients required PMV.^6^ Based on this policy, PMV patients are required to be transferred to a respiratory care center (RCC) after an ICU stay of 21 days, and to respiratory care ward after an RCC stay of 42 days.^6^

Since the introduction of IPP in Taiwan, the epidemiology of PMV patients had been changed.^7,8^ In a national population-based study, Hung et al found that new cases of PMV were significantly increased between 1998 and 2004, and the patients >85 years had highest age-specific incidence rate of PMV.^9^ Although the United States and Canada showed similar trends on age-specific incidence (ie patient's age is proportional to the incidence),^1,2,10–12^ the incident rate of PMV among these very elderly patients was much higher than that previously reported in the United States.^12^ In fact, these very elderly patients are near the end of their life, and most of them prefer preserving quality of life to prolonging their lives.^13,14^ Consequently, this specific population may be reluctant to accept unnecessary life-sustaining therapies such as the use of PMV.^15^ Therefore, studies investigating the outcomes and prognostic factors among the very elderly patients requiring PMV should be warranted. Once the outcome can be fully understood and the prognosis can be accurately predicted, physicians can help patients and their families make the best choice regarding the use of PMV. However, the information on the outcomes and prognosis in the very elderly patients requiring PMV is limited.^16^ The aims of this study were to investigate the outcomes of the very elderly patients requiring PMV and to identify risk factors associated with patients’ mortality.

## METHODS

### Patients and Hospital Setting

Chi Mei Medical Center is a 1288-bed tertiary medical center containing 110 intensive care unit (ICU) beds. The number of beds of RCC was 20 initially and then decreased to 16 since the addition of isolation rooms within RCC in 2011. RCC is responsible for caring ICU patients who are experiencing MV weaning difficulties. Patients eligible for RCC transfer met the following criteria: hemodynamic stability without using vasoactive agents, stable oxygenation, no acute liver or kidney failure, no more need of surgical intervention in the near future, and the attending physicians in ICU believed that the patient may benefit from RCC admission. Between 2006 and 2014, all of the very elderly patients of age 80 or more transferred to the RCC were identified, and patients were included in this study if they used MV for >3 weeks. The data were collected on a routine basis and the analysis was carried out retrospectively. Therefore, no informed consent was required and it was specifically waived by Institution Review Board. Ethics approval was obtained from Institution Review Board of Chi Mei Medical Center.

### Variable Measured

The medical records of all the very elderly patients with PMV were retrospectively reviewed and the following information was collected: age, gender, the category of previous ICU, length of ICU and RCC stay, the diagnosis of RCC admission, duration of MV use in ICU and RCC, Acute Physical and Chronic Healthy Evaluation II (APACHE II) score on ICU and RCC admission, serum album, blood urea nitrogen (BUN) level, serum creatinine, and comorbidities, including coronary artery disease, congestive heart failure, chronic obstructive pulmonary disease, interstitial lung disease, chronic kidney disease, diabetes mellitus, hyperlipidemia, stroke, and cancer. We used in-hospital mortality as the outcome measurement and the deaths caused by any reasons during hospitalization were counted.

### Statistical Analysis

Continuous variables are expressed as means ± standard deviations. The chi-square test or 1-way analysis of variance was used as appropriate to compare each variable/category. A multivariate logistic regression model was used to identify risk factors related to mortality. All statistical analyses were conducted using the statistical package SPSS for Windows (Version 19.0, SPSS, Chicago, IL), and a *P* value < 0.05 was considered to show statistical significance.

## RESULTS

### Demographic Characteristics

**FIGURE 1:**
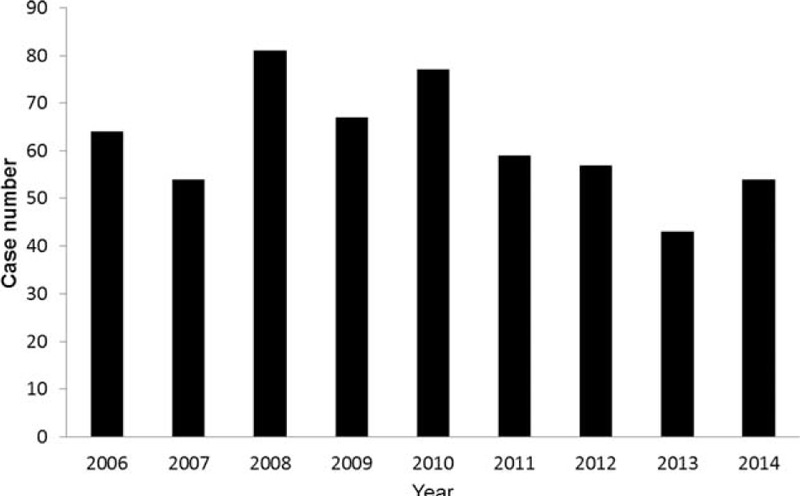
Annual cases of the very elderly requiring prolonged mechanical ventilation.

During the study period, a total of 510 patients were identified and their demographic characteristics were summarized in Table 1 and annual cases of the very elderly requiring prolonged mechanical ventilation were shown in Figure 1. The mean age of the patients was 84.3 ± 3.3 years, and it ranged from 80 to 96 years. Male comprised most of the patients (n = 269, 52.7%), and most of the patients transferred to RCC from medical ICU (n = 357, 70.0%). Pulmonary infection was the most common diagnosis (n = 283, 55.5%), following by neuromuscular disease (n = 79, 15.5%), and decompensated heart disease (n = 58, 11.4%). The APACHE II scores on ICU and RCC admission were 20.6 ± 7.2 and 17.6 ± 6.0, respectively. At least 1 comorbidity was found in 419 (82.2%) patients. Metabolic dysfunction, neurologic diseases, and cardiovascular diseases were the 3 most common comorbidities. In addition, 49 patients (9.6%) required maintenance hemodialysis during hospitalization. Laboratory examinations showed that serum BUN, albumin and creatinine levels were 42.1 ± 27.8 mg/dL, 2.6 ± 0.5 g/dL, 1.4 ± 1.3 mg/dL, respectively. Although we compared the clinical features between octogenarians (aged 80–89) and nonagenarians (aged ≥ 90), no significant differences of gender, disease severity, diagnosis, dialysis, laboratory examinations, comorbidities, and outcomes were found between them (Table 1).

**TABLE 1:**
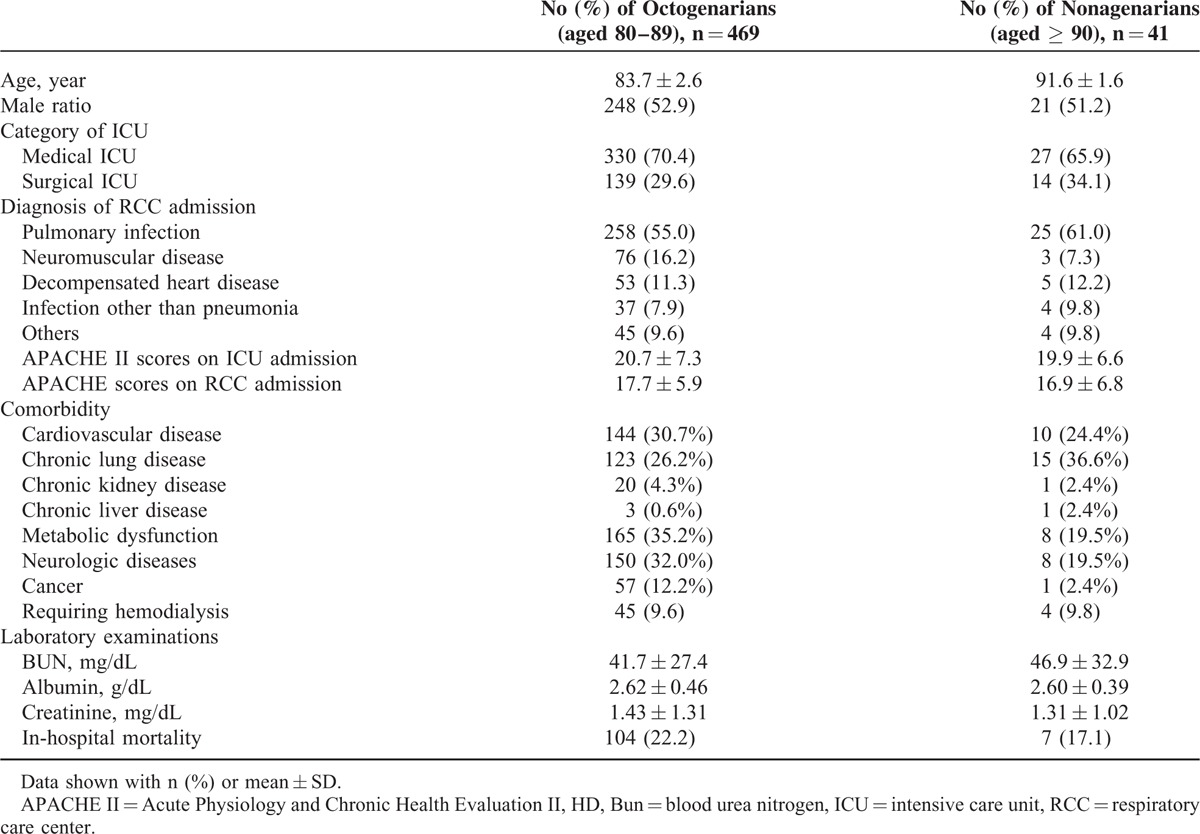
Demographic Characteristics of 510 Very Elderly Patients Required Prolonged Mechanical Ventilation

### Outcome Analysis

**FIGURE 2:**
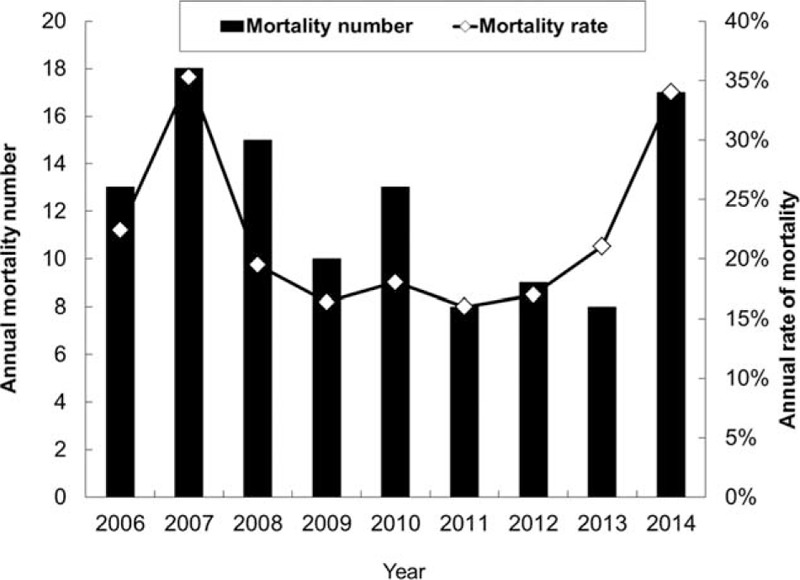
Annual case number and rate of mortality.

Overall, there were a total of 111 deaths, and the in-hospital mortality rate was 21.8% (Figure 2). Table 2 showed prognostic factors associated with in-hospital mortality. In the multivariate analysis, patients who had higher severity on RCC admission (APACHE II score ≥ 15) (odds ratio [OR], 2.30, 95% confidence interval [CI], 1.36–3.90), and lower albumin level (albumin ≤ 2 g/dL) (OR, 3.92, 95% CI, 2.17–7.01) were more likely to have significant in-hospital mortality (*P* < 0.05). In contrast, the outcome was not found to be significantly associated with older age, male gender, comorbidities, the requirement of hemodialysis, and surgical patients.

**TABLE 2:**
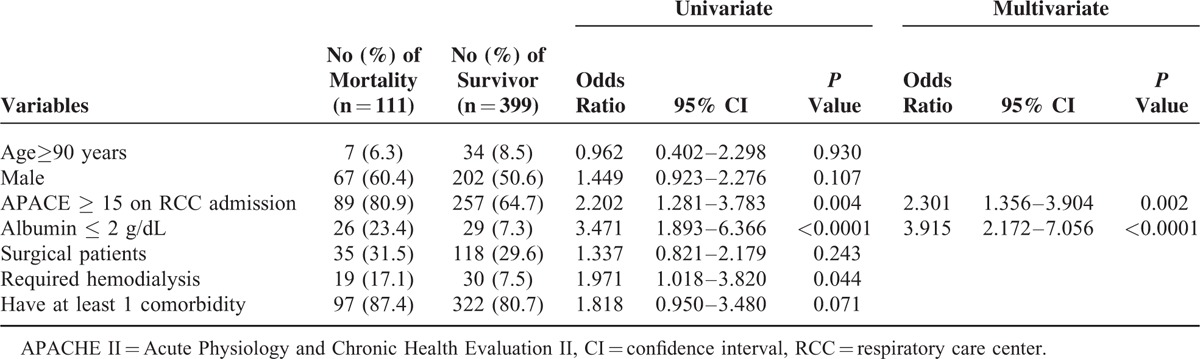
Risk Factors Associated With In-Hospital Mortality Determined by Using Logistic Regression

## DISCUSSION

To our knowledge, this is the first study to investigate the outcomes and prognostic factors of the very elderly patients requiring PMV in a single RCC. Among 510 PMV patients with the mean age of 84.3 years, a total of 111 cases died and the overall in-hospital mortality rate was 21.8%. In addition, we observed a reduced mortality rate from 35.3% in 2007 to 16.0% in 2011, which may be due to the improvement of care. However, we also found an increasing trend of mortality rate from 17.0% to 34.0% between 2012 and 2014. It may be explained by the avoidance of futile medical care and the implementation of palliative care for selected patients, which those polices were encouraged in our hospital since 2012. In the United States, a multicenter study^17^ showed that the rate of in-hospital mortality was 28% (n = 71) of 260 PMV patients with mean age of 55 years. In Brazil, a multicenter study^18^ of 218 PMV patients with mean age of 66.6 years showed that the rate of hospital death was 65% (n = 142). In Wu et al's study of 1212 PMV patients with mean age of 73 years in Taiwan,^19^ the rates of in-hospital mortality were 30.8% and 44.9% among 985 tracheostomy and 227 translaryngeal intubated patients, respectively. In the recent review,^20^ pool mortality at hospital discharge was 29% (95% CI 26–32), and in-hospital mortality was higher in the United States than in non-US countries for postacute care hospitals (31% vs 18%). Despite the disease severity and study designs may be different between our study and the above studies,^17–20^ the outcomes of very elderly patients in this study were not worse than previous studies.^17–20^ In fact, 1 recent study^16^ of 540 PMV patients aged 65 and older in the United States had shown that age was not the dominant factor in predicting outcomes. Another explanation for the relative lower mortality in this specific group in our study is that all of these very elderly patients who can be transferred from ICU to RCC should meet strict criteria after survived the critically ill stage in ICU. Therefore, the difference may be partly due to the high selectivity of patients. Combining with the above studies and our investigation, the results indicated that age itself should not be the only determinant for the outcomes of PMV patients. Even for the PMV patients aged 80 years or more, their in-hospital mortality is not higher than other age-specific groups.

In this study, we identified 2 risk factors associated with in-hospital mortality. These 2 factors are APACHE ≥ 15 and albumin ≤ 2 g/dL. Both of these 2 risk factors may indicate more severe clinical conditions of the patients. In Carson et al's study in a medical center in the United States,^21^ 4 independent predictors of mortality among PMV patients, the requirement hemodialysis and vasopressor, thrombocytopenia, and age ≥ 50 years, were identified. In Taiwan, Lu et al found that neoplasm, renal failure, shock, septicemia, and nonalcoholic liver disease are significantly associated with lower survival among PMV patients by using the National Health Insurance (NHI) system and governmental data on death registry.^22^ However, the information regarding disease severity, APACHE II scores, and actual clinical data such as albumin, are lacking in the NHI database. Therefore, Lu et al^22^ could not find similar risk factors such as APACE ≥ 15 or albumin ≤2 g/dL as those presented in this work. Together with Carson's,^21^ Lu's^22^ and our study in univariate analysis, renal failure, or the requirement of hemodialysis was found to be the common risk to PMV patients’ mortality. This finding is consistent with another nationwide population-based study^23^ in Taiwan, that a significant association was noted between renal function status and the survival of patients with PMV.

For the very elderly patients admitted to ICU, mechanical ventilation may be applied for life saving in the critically ill condition. However, as well as the prolonged use of MV, patients, families, and physicians should consider possible failures of this life-sustaining intervention and the need for palliative care.^24^ In this kind of situation, all of the members who can join decision-making need accurate prognostic information to evaluate whether the goal for caring this very elderly patient needs to be changed or not. Our study may provide useful information to help physicians discuss the appropriate timing of palliative care for end-of-life with all of the persons of interests.

Our study has several limitations. First, only in-hospital mortality was measured in the study. We did not assess the outcomes of the patients after discharge and cannot assay the long-term outcome such as 1-year survival rate. In addition, we neither evaluated the quality of life among study subjects, patient's preference for PMV nor conducted economic analysis of this proposal implemented in our hospital. Further study is still warranted to clarify these issues. Second, our findings are based on a single institution, and the study subjects are highly selective as the patients were transferred from ICU to RCC. Therefore, it may not be suitable to generalize our study results to other hospital or countries. However, our study is the first one that focuses on this issue and enrolls large numbers of patients for a long period. Currently, our findings still remain representative of this specific population.

In conclusion, the in-hospital mortality rate of the very elderly PMV patients is about one-fifth, which has no statistical difference between octogenarians and nonagenarians based on strict RCC admission criteria. However, poor outcomes in this specific population were found to be associated with higher APACHE II score and lower albumin level.
